# Environmental endocrine disruptor-induced mitochondrial dysfunction: a potential mechanism underlying diabetes and its complications

**DOI:** 10.3389/fendo.2024.1422752

**Published:** 2024-08-15

**Authors:** Kunhui He, Rumeng Chen, Shuling Xu, Yining Ding, Zhu Wu, Meihua Bao, Binsheng He, Sen Li

**Affiliations:** ^1^ The 1^st^ Affiliate Hospital of Changsha Medical University, Changsha Medical University, Changsha, China; ^2^ Hunan Key Laboratory of the Research and Development of Novel Pharmaceutical Preparations, School of Pharmaceutical Science, Changsha Medical University, Changsha, China; ^3^ School of Life Sciences, Beijing University of Chinese Medicine, Beijing, China; ^4^ The Hunan Provincial Key Laboratory of the TCM Agricultural Biogenomics, Changsha Medical University, Changsha, China

**Keywords:** endocrine disruptor, type 2 diabetes mellitus, diabetic complications, mitochondrial dysfunction, reactive oxygen species

## Abstract

Diabetes and its complications significantly affect individuals’ quality of life. The etiology of diabetes mellitus and its associated complications is complex and not yet fully understood. There is an increasing emphasis on investigating the effects of endocrine disruptors on diabetes, as these substances can impact cellular processes, energy production, and utilization, ultimately leading to disturbances in energy homeostasis. Mitochondria play a crucial role in cellular energy generation, and any impairment in these organelles can increase susceptibility to diabetes. This review examines the most recent epidemiological and pathogenic evidence concerning the link between endocrine disruptors and diabetes, including its complications. The analysis suggests that endocrine disruptor-induced mitochondrial dysfunction—characterized by disruptions in the mitochondrial electron transport chain, dysregulation of calcium ions (Ca^2+^), overproduction of reactive oxygen species (ROS), and initiation of signaling pathways related to mitochondrial apoptosis—may be key mechanisms connecting endocrine disruptors to the development of diabetes and its complications.

## Introduction

According to the most recent epidemiological survey data from 2021, an estimated 537 million individuals were diagnosed with diabetes, 541 million had impaired glucose tolerance, and diabetes was associated with 6.7 million deaths (1). Given the continual increase in the diabetic patient population, projections suggest that the global diabetic population will rise to 783 million by the year 2045 (1). Effective management of blood glucose levels and clinical outcomes for diabetic patients remains suboptimal, often resulting in complications that affect multiple organs, including the eyes, kidneys, heart, and peripheral nerves (2–10). Diabetes and its complications pose a significant burden (11, 12). Diabetes has various known risk factors (13–16), and methods for diabetes prevention and treatment have been investigated and developed (17–20). However, the current understanding of the pathogenesis of diabetes and its associated complications remains incomplete, which hinders the development of effective clinical prevention and treatment strategies.

Endocrine disruptors encompass a wide array of exogenous chemicals, comprising approximately 1,000 types, including substances such as nonylphenol, pesticides, metals, fungicides, and others (21). These substances are ubiquitous in human living environments, present in items such as furniture, paint, flooring, electronic devices, toys, food packaging, bottled beverages, cosmetics, receipts, clothing, food, contact lenses, and dental sealants. As modern civilization advances, the growing demand for novel chemicals has heightened human exposure to endocrine-disrupting chemicals (EDCs). It is estimated that 147 of these substances may persist in the environment or are produced in large quantities, thereby posing a significant risk to human health (22, 23). A growing body of research indicates that EDCs increase the risk of various metabolic diseases, including obesity (24–31), diabetes and its complications (32–43), and metabolic syndrome (44–51) . For example, studies have demonstrated that long-term daily exposure to EDCs, even at concentrations below the established tolerance thresholds for individual substances, can significantly increase the risk of diabetes in both women and men (52). However, these studies did not explore the specific mechanisms that underlying the influence of endocrine disruptors on diabetes and its complications.

The increasing number of studies establishing a connection between endocrine disruptors and diabetes, along with its complications, via mitochondrial dysfunction, may be attributed to the interaction between mitochondria and hormone secretion, the generation of reactive oxygen species (ROS), and their effects on metabolic homeostasis (53–57). Mitochondria are essential cellular components that play a crucial role in energy metabolism (58). Adenosine triphosphate (ATP) is primarily produced in the mitochondria through the coupling of the tricarboxylic acid cycle (TCA) with oxidative phosphorylation (OXPHOS) (59, 60). Disruption in this process caused by endocrine disruptors can result in mitochondrial damage and dysfunction, leading to increased production of superoxide free radicals and ultimately raising the risk of developing diabetes (61). Nonetheless, further exploration is required to elucidate the exact role of endocrine disruptors in initiating diabetes and its complications through mitochondrial dysfunction. This study will review the most recent literature from the past five years, beginning with an overview of the latest evidence regarding various endocrine disruptors associated with the onset of diabetes and its complications. Following this, the study will elucidate the mechanisms through which mitochondrial dysfunction contributes to the development of diabetes induced by endocrine disruptors. Lastly, it will outline how mitochondrial dysfunction, triggered by endocrine disruptors, leads to diabetic complications. **Table 1** summarizes the literature cited in this review, primarily from clinical studies.

**Table 1:** Endocrine disruptors and their relationship with diabetes and its complications in clinical studies.

Year	Title	Study Type	Endocrine disruptors	Biological Sample	Population		Follow-up period	Findings	Ref
				Matrix	Country	Size			
2019	Novel insights of elevated systemic levels of bisphenol-A (BPA) linked to poor glycemic control, accelerated cellular senescence and insulin resistance in patients with type 2 diabetes	cross-sectional	BPA	Serum	India	60	Not available	Levels of BPA were markedly increased and exhibited a positive correlation with inadequate glycemic control and insulin resistance.	(62)
2019	Exposure to Bisphenol A and Bisphenol S and Incident Type 2 Diabetes: A Case-Cohort Study in the French Cohort D.E.S.I.R.	case-cohort	BPA and BPS	Urine	French	755	9 years	Positive correlation between exposure to BPA and BPS and the prevalence of type 2 diabetes.	(63)
2019	Exposure to bisphenol A and diabetes risk in Mexican women	case-control	free BPA	Urine	Mexico	404	Not available	Positive significant association between urinary free BPA and diabetes	(64)
2020	Human exposure to bisphenol A through dietary sources and development of diabetes mellitus: a cross-sectional study in Pakistani population	cross-sectional	BPA	Urine	Pakistan	400	Not available	A significant correlation was found between exposure to BPA and risk factors for diabetes mellitus.	(65)
2021	Association between type 2 diabetes and exposure to chlorinated persistent organic pollutants in Algeria: A case-control study.	case-control	Chlorinated persistent organic pollutants	Plasma	Algeria	361	Not available	Environmental exposure to specific persistent organic pollutants is linked to a heightened risk of type 2 diabetes.	(66)
2019	Exposure to pesticides and the prevalence of diabetes in a rural population in Korea	cross-sectional	Pesticides	Questionnaires	Korea	2559	Not available	After adjusting for covariates, there was an association between pesticide exposure and the risk of diabetes.	(67)
2020	Pesticide exposure and diabetes mellitus in a semi-urban Nepali population: a cross-sectional study.	cross-sectional	Pesticide	Questionnaires	Nepal	2310	Not available	No correlation observed between pesticide exposure and diabetes mellitus in the low-exposure population.	(68)
2019	Metabolite of the pesticide DDT and incident type 2 diabetes in urban India.	nested case-control	Pesticide DDT	Plasma	India	516	Not available	No significant association observed between p, p-DDE and type 2 diabetes.	(69)
2023	Relationships Between Urinary Metals and Diabetes Traits Among Mexican Americans in Starr County, Texas, USA.	cross-sectional	Metal	Urine	America	414	Not available	Arsenic, molybdenum, copper, and this specific metal combination are linked to modified glucose homeostasis parameters in non-diabetic individuals.	(34)
2020	Association between triclocarban and triclosan exposures and the risks of type 2 diabetes mellitus and impaired glucose tolerance in the National Health and Nutrition Examination Survey (NHANES 2013-2014)	cross-sectional	Triclocarban and triclosan	Urine	America	900	Not available	Positive association was identified between triclocarban exposure and type 2 diabetes in women.	(33)
2020	Aryl-hydrocarbon receptor binding and the incidence of type 2 diabetes: the Brazilian Longitudinal Study of Adult Health (ELSA-Brasil).	longitudinal Study	Persistent organic pollutants	Serum	Brazil	543	2 years	Elevated aryl hydrocarbon receptor ligand-induced luciferase bioactivity and reduced intracellular ATP content in serum-incubated samples were correlated with the onset of diabetes.	(70)
2019	Dietary exposure to brominated flame retardants and risk of type 2 diabetes in the French E3N cohort	prospective cohort	Brominated flame retardants	Food	French	71,415	19 years	There was a correlation between dietary exposure to brominated flame retardants and the risk of type 2 diabetes.	(71)
2023	Exposure to novel brominated and organophosphate flame retardants and associations with type 2 diabetes in East China: A case-control study	case-control	Novel brominated and organophosphate flame retardants	Serum	China	344	Not available	Exposure to emerging brominated and organophosphate flame retardants may contribute to the development of type 2 diabetes.	(32)
2019	Perfluoroalkyl substances and risk of type II diabetes: A prospective nested case-control study	nested case-control	Perfluoroalkyl substances	Plasma	Sweden	248	10 years	A general inverse association was observed between Perfluoroalkyl substances and the risk of type 2 diabetes.	(72)
2021	Associations of urinary concentrations of phthalate metabolites, bisphenol A, and parabens with obesity and diabetes mellitus in a Korean adult population: Korean National Environmental Health Survey (KoNEHS) 2015–2017	cross-sectional	Phthalate metabolites, BPA, and parabens	Urine	Korea	3782	Not available	Elevated urinary concentrations of ethyl paraben and BPA are linked to diabetes mellitus.	(73)
2020	Historical exposure to non-persistent environmental pollutants and risk of type 2 diabetes in a Spanish sub-cohort from the European Prospective Investigation into Cancer and Nutrition study	cohort	Non-persistent environmental pollutants	Serum	Spain	670	23 years	Individuals in the propyl paraben quartile (0.53-9.24 ng/ml) exhibited a statistically significant elevated risk of type 2 diabetes.	(35)
2019	Association between urinary parabens and gestational diabetes mellitus across prepregnancy body mass index categories.	cross-sectional	Parabens	Urine	China	696	Not available	Moderate levels of propyl paraben and paraben-estrogen activity were significantly linked to a higher prevalence of gestational diabetes mellitus in overweight or obese pregnant women.	(74)
2019	Associations of Perfluoroalkyl and Polyfluoroalkyl Substances With Incident Diabetes and Microvascular Disease	prospective cohort	Perfluoroalkyl and Polyfluoroalkyl Substances	Plasma	America	957	15 years	Both Sm 2-perfluoromethylheptane sulfonic acid and N-ethyl perfluoroane sulfonamide acetic acid are linked to diabetic microvascular disease.	(36)
2019	Bisphenol A, Chlorinated Derivatives of Bisphenol A and Occurrence of Myocardial Infarction in Patients with Type 2 Diabetes: Nested Case-Control Studies in Two European Cohorts	nested case-control	BPA, Chlorinated Derivatives of BPA	Urine	French and Germany	292	Not available	The relationship between urinary concentrations of BPA and chlorinated derivatives of BPA and myocardial infarction events in patients with type 2 diabetes.	(75)

## Endocrine disruptors and diabetes mellitus

Endocrine disruptors are primarily classified into two groups: persistent endocrine disruptors and non-persistent endocrine disruptors. Studies consistently shown that among persistent endocrine disruptors, the relationship between persistent organic pollutants (POPs) and type 2 diabetes (T2D) is notably significant (76). Major POPs include organochlorine pesticides (OCPs) such as dichlorodiphenyldichloroethylene (DDE) and its metabolite chlordane, brominated flame retardants (BFRs), and per- and polyfluoroalkyl substances (PFAS). A prospective study conducted in Brazil enrolled 71 diabetes cases and 472 randomly selected controls. The study assessed AhR ligand bioactivity (AhRL), which reflects the cumulative effect of various POPs, and intracellular ATP levels, indicative of mitochondrial inhibitory substances (MIS). The findings indicated that, in comparison with the control group, the diabetes group exhibited a significant 6.2% reduction in MIS-ATP levels (*P* < 0.0001). In crude analysis, participants with AhRL levels above the median had a higher risk of diabetes (hazard ratio (HR) = 1.85; 95% confidence interval (CI): 1.11-3.06), while those with MIS-ATP levels below median faced an increased diabetes risk (HR = 3.62; 95% CI: 2.06-6.38). Furthermore, individuals with elevated AhRL and low MIS-ATP showed a significantly heightened diabetes risk when compared with those with low AhRL and high MIS-ATP (HR = 8.15; 95% CI: 2.86-23.25), which was slightly attenuated after adjusting for smoking (HR = 6.9; 95% CI: 2.4-20) (70). This indirectly suggests that over an extended period exposure to POPs may lead to the development of diabetes. Additionally, findings from a case-control study conducted in Algeria indicated that, after adjusting for known risk factors for T2D in Algeria, environmental exposure to OCPs could significantly increase the likelihood of developing T2D. Specifically, the study highlighted associations with 4,4′-DDE (odds ratio (OR): 12.58; 95% CI: 4.76-33.26) and hexachlorobenzene (OR: 3.69; 95% CI: 1.90-7.15) (66). Moreover, findings from a meta-analysis study suggested a significantly elevated risk of diabetes associated with increased exposure to the organochlorine insecticide chlordane (77). In addition to OCPs, there is increasing evidence that exposure to organophosphorus pesticides may also increase the risk of T2D (78). Additionally, a cross-sectional study conducted in Korea revealed significant associations between pesticide exposure variables and the prevalence of diabetes in the Korean population, after adjusting for potential risk factors such as age, gender, monthly income, and education level. These variables included pesticide use (OR: 1.58; 95% CI: 1.13-2.21), duration of use (>20 years) (OR: 1.51; 95% CI: 1.07-2.14), frequency of use (more than 10 days per year) (OR: 1.53; 95% CI: 1.09-2.15), intensity of exposure (low intensity: OR: 1.55; 95% CI: 1.07-2.24, high intensity: OR: 1.53; 95% CI: 1.06-2.22), and cumulative exposure index (high exposure: OR: 1.54; 95% CI: 1.03-2.30). This association was particularly pronounced among individuals who were overweight or obese (67). Conversely, no correlation was observed between pesticide exposure and T2D in study populations predominantly comprising farmers in Nepal and India (68, 69). Thus, the current data on pesticide-induced diabetes lack consistency, which may stem from variations in experimental designs. In studies from Korea and Nepal, pesticide exposure was assessed solely through questionnaires, which are vulnerable to recall bias. Conversely, the study from India evaluated pesticide exposure using biomarkers, specifically measuring blood p,p-DDE concentration once, which may not accurately reflect lifetime exposure. Moreover, the use of a single-pesticide model in the study does not eliminate the potential confounding effects of other pesticide residues. BFRs are prominent POPs. Findings from a French E3N cohort study suggested that dietary exposure to BFRs might increase the risk of developing T2D (71). Moreover, a case-control study conducted in China, involving 172 individuals with T2D and 172 controls, indicated that exposure to novel BFRs and organophosphorus flame retardants may contribute to the development of T2D (32). PFAS constitute a substantial category of highly fluorinated organic compounds known for their exceptional persistence. A conditional logistic regression from a prospective nested case-control study found that, after adjusting for gender, age, sample year, diet, and body mass index, long-term exposure to PFAS was potentially associated with reduced insulin resistance in individuals without T2D (72).

Non-persistent endocrine disruptors mainly include bisphenol A (BPA), heavy metals, and fungicides. BPA is a primary component of polycarbonate plastics, and studies have detected BPA in over 95% of the global population (79), making it one of the most widespread endocrine disruptors. Several observational studies have shown a significant correlation between exposure to BPA and insulin resistance, disrupted glucose homeostasis, and the development of T2D in diverse racial or population groups (62–65). Parabens are antimicrobial preservatives frequently employed in consumer goods and are classified as non-persistent environmental contaminants. This group encompasses methylparaben, ethyl paraben, propylparaben, and butylparaben. A cross-sectional study conducted in Korea identified ethyl paraben as potential risk factors for T2D after adjusting for covariates (73). Additionally, a cohort study conducted in Spain (n=670) assessed exposure through chemical analysis of serum samples collected at recruitment. After a median follow-up of 23 years, 182 cases (27%) of T2D were diagnosed. The results of the Cox proportional hazards model showed that individuals with propylparaben levels between 0.53 and 9.24 ng/ml had a statistically significant increased risk of T2D (HR = 1.668, *P* = 0.012) (35). Nevertheless, a study conducted in China did not observe a relationship between paraben exposure and gestational diabetes mellitus in the overall population (74). A study involving 414 Mexican Americans revealed that elevated levels of urinary arsenic, molybdenum, copper, and a combination of these metals could disrupt insulin-glucose homeostasis and fasting insulin levels (34). In another cross-sectional study involving 900 American participants, after adjusting for potential confounders, exposure to fungicides (specifically triclocarban) was found to elevate the risk of T2D in women (OR: 1.79; 95% CI: 1.05–2.05) (33). Studies on non-persistent endocrine disruptors, despite using exposure assessments like biomarkers such as blood or urine measurements to mitigate recall bias from questionnaire surveys, are inherently limited by their observational nature. They cannot definitively rule out the influence of other potential confounding factors. EDCs tend to be highly correlated with each other, and individuals are commonly exposed to mixtures of various types, which complicates the precise determination of the independent effects of each compound. Mendelian randomization (MR) analysis is an epidemiological method that uses genetic variation associated with the exposure being studied as a tool to reduce biases from confounding factors and address reverse causation (80–83). Thus, future research could benefit from MR studies to explore causal relationships between EDC exposure and health outcomes.

Recent research on the relationship between EDCs and diabetes has increased, yet determining which type of EDC has a greater impact on diabetes remains challenging. This difficulty arises not only from the vast variety of EDCs and their widespread presence in human environments (84), but also from the fact that EDCs rarely act independently. They often work synergistically and cumulatively, with long latency periods of exposure (21). Diseases caused by EDCs may not become apparent during the exposure period but might manifest in adulthood or old age. Moreover, the adverse effects of EDCs can be transmitted to subsequent generations, even if those generations are not directly exposed (85). These factors collectively increase the complexity of studying the effects of EDCs on human health.

## The role of mitochondrial dysfunction in mediating the impact of endocrine disruptors on diabetes

Diabetes is well recognized as a disease stemming from pancreatic β-cell dysfunction influenced by various factors, often involving ROS generation, primarily originating from mitochondria. Mitochondria serve a pivotal role in ATP production via OXPHOS (86). Under normal circumstances, acetyl-CoA derived from lipid and glucose metabolism enters the mitochondrial matrix to engage in the TCA cycle. During this process, substrates undergo oxidation, yielding CO_2_, flavin adenine dinucleotide (FADH2), and nicotinamide adenine dinucleotide (NADH) (59, 86–88). Electrons from NADH and FADH2 are accepted by Complexes I and II, respectively, and subsequently transferred through ubiquinone (Q) and cytochrome c (C) to Complexes III and IV. Molecular oxygen acts as the final electron acceptor at Complex IV, converting to water. Throughout this redox process, Complexes I, II, and IV facilitate proton pumping from the matrix into the intermembrane space, thereby establishing an electrochemical gradient (membrane potential) critical for ATP synthesis (86, 89). The integrity of this mitochondrial electron transport chain is essential for sustained electron transfer. Disruption of this chain can lead to heightened ROS production, diminished ATP levels, disturbances in calcium ion (Ca^2+^) homeostasis, alterations in membrane permeability, structural impairments in mitochondria, and potential cellular demise (90–92).

**Figure 1:**
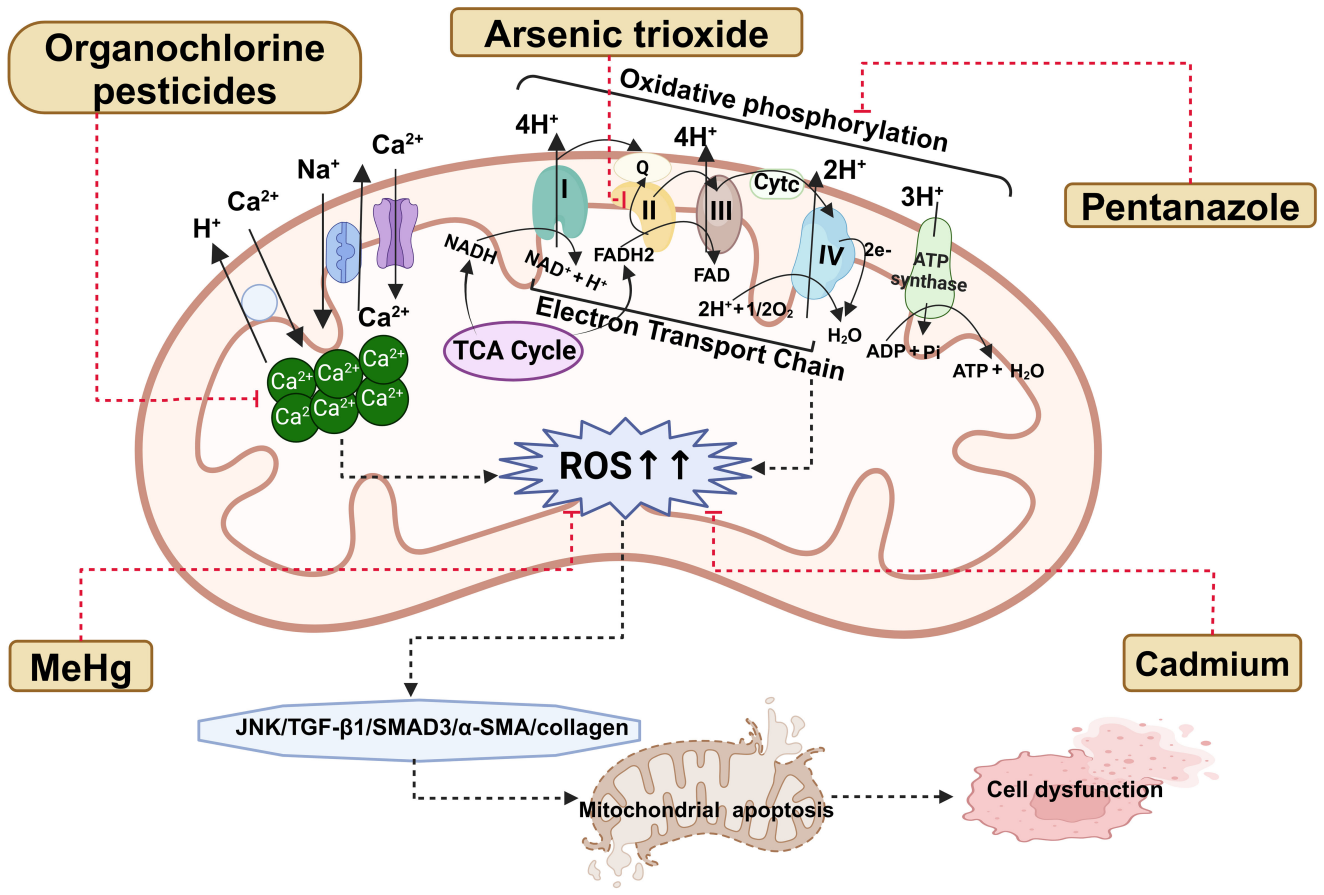
The pathways linking endocrine disruptors to diabetes and its complications through mitochondrial dysfunction are discussed within this review. ROS, reactive oxygen species; TCA, tricarboxylic acid cycle; ADP, adenosine diphosphate; ATP, Adenosine triphosphate; H^+^, Hydrogen ion; Ca^2+^, calcium ions; Na^+^, sodium ion; I, complex I; II, complex II; III, complex III; IV, complex IV; Q, ubiquinone; Cytc, cytochrome c; FAD, flavin adenine dinucleotide; FADH2, flavin adenine dinucleotide; NADH, nicotinamide adenine dinucleotide; NAD^+^, nicotinamide adenine dinucleotide; O_2_, oxygen; H_2_O, Water.

Ca^2+^ acts as a signaling molecule that enhances OXPHOS, including the TCA cycle and the subsequent electron transport chain. An animal study found that when zebrafish liver mitochondria were exposed to OCP mixture, the mitochondria in the exposed group absorbed Ca^2+^ more quickly than those in the unexposed group. Additionally, the activity of mitochondrial complex III was reduced (93). This suggests that the OCP mixture can promote calcium influx by regulating mitochondrial calcium channels, leading to mitochondrial swelling. Consequently, this affects OXPHOS and disrupts the electron transport chain, causing the mitochondrial membrane potential to collapse (94), limiting ATP production, and resulting in a significant accumulation of ROS, ultimately leading to cell death (95). Additionally, another animal study exposed mitochondria isolated from pre-diabetic rats to a combination of low concentrations of arsenic trioxide (ATO) (IC25 = 40 μM) and hyperglycemic conditions (glucose levels at 20, 40, 80, and 160 mM) or pyruvate (PYR, 20,40,80,160 mM). The results showed that when mitochondria were exposed to various concentrations of glucose alone or in combination with a lower dose of ATO, there were no adverse effects on the mitochondria. However, exposure to a higher concentration of PYR(≥40mM) with ATO (40 μM) resulted in decreased mitochondrial dehydrogenase activity (complex II), increased mitochondrial ROS (mtROS) production, lipid peroxidation, glutathione depletion, and ultimately, mitochondrial membrane damage and swelling (96). In an experiment with RIN-m5F cells, it was found that the activation of ROS-activated JNK signaling is a key mechanism in methylmercury (MeHg)-induced mitochondria-dependent apoptosis. After treatment with MeHg (2μM), there was mitochondrial dysfunction, evidenced by the depolarization of the mitochondrial membrane potential and increased cytochrome c (C) release. Apoptotic responses included elevated protein expression levels of cleaved caspase-3 and -7, as well as the upstream caspase-9, annexin V-Cy3 binding, and an increased pro-apoptotic (Bax, Bak, p53)/anti-apoptotic (Bcl-2) mRNA ratio. Additionally, there was JNK phosphorylation, increased protein expression of ERK1/2 and Akt, and a significant increase in intracellular ROS production (97). Another *in vitro* experiment using the MIN6 cell model found that cadmium treatment induces mitochondrial dysfunction. Following cadmium treatment, there was a significant increase in intracellular ROS and mtROS levels, a marked decrease in ATP production, significant depolarization of the mitochondrial membrane potential, a reduction in mitochondrial DNA copy number, and suppression of the expression of mitochondrial transcription factor A (TFAM) (98). In conclusion, irrespective of the specific EDCs individuals are exposed to and the signaling pathways involved, it is evident that these disruptions can instigate mitochondrial apoptosis, resulting in β-cell dysfunction, and ultimately contributing to the onset of diabetes. **Figure 1** encapsulates the primary theme of the review, highlighting the interconnections between endocrine disruptors, diabetes, and mitochondrial dysfunction.

## Endocrine disruptors and diabetic complications

Diabetes complications primarily encompass microvascular lesions affecting organs such as the kidneys and retina, as well as macrovascular lesions involving the heart and peripheral blood vessels. Currently, there is limited research directly investigating the impact of endocrine disruptors on diabetic complications. Most studies primarily focus on investigating the direct harmful effects of endocrine disruptors on organs such as the kidneys, retina, and heart. In a prospective cohort study conducted in the United States, 957 non-diabetic adults with elevated fasting and post-load glucose levels were enrolled. Participants were randomized into either an intensive lifestyle intervention focusing on diet, physical activity, and behavioral correction, or a placebo medication group. Plasma concentrations of six PFAS compounds were measured at baseline and after 2 years of follow-up. After a 15-year follow-up period, the occurrence of microvascular diseases was assessed, adjusting for potential risk factors. Results from a multivariable logistic regression model indicated that N-ethyl-perfluorooctane sulfonamido acetic acid (OR 1.17, 95% CI 1.05-1.31) and perfluorodimethylhexane sulfonic acid (OR 1.18, 95% CI 1.04-1.35) may increase the risk of microvascular disease (36). An observational study carried out in America identified a link between elevated levels of dioxins/furans and renal insufficiency (99). Furthermore, a distinct study suggested that prenatal exposure to air pollution could have adverse effects on fetal kidney function, while exposure to green spaces might confer a beneficial impact (100). Moreover, a review concentrating on the influence of chemical agents on the retina underscored the retinal toxicity associated with insecticides such as chlorpyrifos, thiamethoxam (THIA), and lefenuron, along with fungicides like triphenyltin and thiamine. These substances were observed to cause structural alterations in retinal cells and have the potential to result in serious conditions like retinal detachment, fundus hemorrhage, and pupil constriction (101). A recent systematic review unveiled that increased levels of circulating OCPs and polychlorinated biphenyls are associated with a heightened risk of coronary heart disease (102). Additionally, an observational study carried out in Honolulu, America, showcased a positive association between age-adjusted incidence of cardiovascular disease (CVD) and elevated levels of pesticide exposure during the initial 10 years of follow-up (HR = 1.46, 95% CI = 1.10-1.95). This relationship retained statistical significance even after adjusting for other CVD risk factors (HR = 1.42, 95% CI = 1.05-1.92) (103).

Diabetic nephropathy (DN) is recognized as one of the most prevalent microvascular complications of diabetes. BPA, a prevalent endocrine disruptor, has been associated with proteinuria, which may serve as a predictor for the progression of chronic kidney disease (104). Furthermore, an animal study illustrated that chronic exposure to BPA exacerbated renal function impairment in diabetic rats, resulting in hemodynamic disturbances and dysfunction (105). Coronary artery disease is a prevalent complication in diabetes that affects large vessels and stands as the leading cause of mortality among diabetic complications. Two nested case-control studies were independently conducted across European cohorts to investigate the association between BPA exposure and myocardial infarction (MI) occurrence in patients with T2D. Each MI case identified during the study period was matched with a control from the same cohort based on age, sex, and individual cardiovascular history. The analysis focused on baseline urinary BPA levels, which indicated exposure rates of 31% in the ESTHER cohort and 18% in the SURDIAGENE cohort. A meta-analysis combining both studies revealed a significant correlation between detectable urinary BPA levels and MI occurrence, yielding an adjusted OR of 1.97 (95% CI: 1.05-3.70, *P* = 0.04) (75). The aforementioned studies indicate that prolonged exposure to BPA may contribute to damage in diabetic vasculature. However, diabetes complications carry significant morbidity and mortality rates, and given the diverse array of endocrine disruptors, future research could explore the effects of various types of endocrine disruptors on diabetes complications. Additionally, as most of these studies are observational rather than causal investigations, they cannot entirely dismiss the potential confounding effects of factors such as critical periods of exposure during pregnancy and early childhood, as well as the influence of genetic polymorphisms on the outcomes.

## The role of mitochondrial dysfunction in mediating the impact of endocrine disruptors on diabetic complications

An animal experiment suggested that overactivation of renal mitochondrial activity could contribute to metabolic disorders and the development of early DN (106). Moreover, another animal study and cell study showed that cadmium exposure exacerbated kidney injury in diabetic rats by inhibiting autophagy (54). The study revealed that compared to the normal control group, diabetic rats exposed to cadmium exhibited significantly increased levels of malondialdehyde, reduced levels of glutathione (GSH), decreased activity of superoxide dismutase (SOD), decreased activation of catalase (CAT), elevated ROS levels, and an increased number of autophagosomes. Furthermore, the expression of autophagy-related proteins, markers of apoptosis, and fibrosis markers was significantly higher in the exposed group compared to the unexposed group (54). SOD functions by converting superoxide radicals into molecular oxygen and hydrogen peroxide, thus eliminating them. CAT then aids in converting hydrogen peroxide into water and oxygen. GSH plays a role in scavenging ROS (107). When the activity of these enzymes decreases, there will be a notable accumulation of ROS. Subsequent to cadmium chloride exposure, there is a substantial rise in ROS production in renal cells, resulting in oxidative damage to cellular macromolecules, increased lipid peroxidation, protein oxidation, DNA damage, and fragmentation. This cascade activates the transforming growth factor (TGF)-β1/SMAD3/α-SMA/collagen signaling pathway, ultimately leading to renal fibrosis (107). Additionally, another animal study indicated that cadmium chloride exposure triggers renal fibrosis by activating the TGF-β1/mothers against decapentaplegic homolog (Smad)/collagen IV signaling pathway, stimulating proinflammatory mediators, inhibiting the activation of nuclear factor erythroid 2-related factor 2, and enhancing apoptotic signaling (108). An animal study showed that acute exposure to a sublethal dose of THIA led to cell death and structural damage in the eyes of flies (109). Furthermore, in another animal study, female rats were orally administered cypermethrin (CYP) daily from day 7 of gestation until delivery. The sensory retinas of the rats in the CYP subgroup displayed vacuolation in the inner and outer plexiform layers, dilation of hyperemic blood vessels, hyalinization, and disruptions in the photoreceptive layer (110). Mitochondrial dysfunction reduces ATP production and utilization, resulting in heart damage (111, 112). Several studies have documented a strong correlation between mtROS and CVDs (113, 114). An animal study demonstrated that tebuconazole, a commonly used agricultural drug, could accumulate in the heart, leading to a decrease in ATP content in mouse hearts. TFAM plays a crucial role in regulating mitochondrial biogenesis (115), OXPHOS governs the final phases of the eukaryotic mitochondrial respiratory chain (116). The mRNA levels of TFAM and OXPHOS complex subunits were notably reduced in the hearts of mice exposed to the tebuconazole, implying that it impedes mitochondrial biosynthesis and disrupts respiratory chain function, thereby worsening mitochondrial cardiac dysfunction (117). **Figure 1** also depicts the interaction between endocrine disruptors, diabetic complications, and mitochondrial dysfunction.

## Conclusion

Epidemiological evidence indicates that exposure to EDCs increases the risk of diabetes and its complications. Proposed mechanisms suggest that EDCs may induce mitochondrial dysfunction through several pathways, including disruption of the electron transport chain, perturbation of Ca^2+^ homeostasis, increased production of ROS, and activation of mitochondrial apoptotic pathways. However, due to the diverse range of EDCs and their cumulative effects in the human body, isolating the independent impact of a single EDC on disease development poses significant challenges. There is a pressing need for enhanced methods to extract and analyze EDCs effectively. Moreover, since most current studies are observational, they are limited in their ability to fully elucidate the precise mechanisms through which EDCs influence disease development. Future research efforts should incorporate more animal and cellular studies to complement these observational findings and provide deeper insights into these mechanisms.
